# Diagnosis and Management of Type 2 Acute Coronary Syndrome in Patients Without Pre-existing Cardiomyopathy in Centers With and Without Percutaneous Coronary Intervention

**DOI:** 10.7759/cureus.83137

**Published:** 2025-04-28

**Authors:** Victor Fournier, Pierre Nazeyrollas, Corentin Lefebvre, Damien Metz, Laurent Faroux

**Affiliations:** 1 Cardiology, Centre Hospitalier Universitaire (CHU) Robert Debré, Reims, FRA; 2 UFR (Training and Research Unit) Sciences, Université de Reims Champagne-Ardenne, Reims, FRA

**Keywords:** acute coronary syndrome, coronary angiography, emergency reception service, oxygen consumption, troponin, type 2 myocardial infarction

## Abstract

Introduction

Type 2 acute coronary syndrome (ACS) is defined as myocardial infarction caused by an imbalance between myocardial oxygen supply and demand. Although several observational studies have been conducted, they have not led to the development of clear clinical guidelines, partly due to heterogeneous definitions and the absence of consensus regarding diagnostic criteria and management strategies.

Aims

To assess diagnostic practices and management of type 2 ACS in patients without pre-existing heart disease and to analyze these practices based on the availability of coronary angiography at the inclusion center.

Methods

We retrospectively reviewed the records of 25,225 patients who received a troponin assay in the emergency departments of two centers, one with and the other without coronary angiography facilities, from 2018 to 2019. We selected 224 patients without pre-existing heart disease who had type 2 ACS according to objective criteria. The study was designed as a retrospective, descriptive, and comparative analysis. Data on clinical characteristics, diagnostic labeling, and management strategies, including cardiology consultations and ischemic investigations, were collected and analyzed.

Results

The study populations from both centers were similar. Clinicians diagnosed type 2 ACS in only three (1.3%) cases, despite a high rate of cardiology consultations (180, 80.4%). Exploration was performed in 78 (34.8%) patients, revealing significant coronary lesions in 43 (64.2%) cases. In 118 (74.7%) patients without exploration during hospitalization, no rationale was documented. The presence of on-site coronary angiography did not significantly influence the decision to explore (p = 0.067). Exploration decisions were influenced by age (p < 0.001), family history (p = 0.006), ECG presentation (p = 0.034), left ventricular ejection fraction (LVEF) (p = 0.02), and cardiology consultation request (p < 0.001).

Conclusion

Objective criteria allowed the selection of a homogeneous type 2 ACS population. The diagnosis is rarely made, highlighting the need for increased awareness among emergency physicians and cardiologists. Integrating objective criteria into clinical guidelines could be considered. Given the high rate of underlying coronary lesions, patients with type 2 ACS and no pre-existing heart disease should be prioritized for coronary exploration.

## Introduction

Myocardial infarction is defined by the European Society of Cardiology (ESC) [1] as an increase or decrease in cardiac markers, with at least one of the values above the 99th percentile of the reference population, associated with a sign of myocardial ischemia. This broad definition goes beyond the acute coronary occlusion, the type 1 acute coronary syndrome (ACS), and creates new entities such as type 2 ACS, which is secondary to an imbalance in the oxygen (O2) supply/demand of the myocardium. The diagnosis and management of this pathology are important since studies have shown a worse prognosis than type 1 ACS [2], with mortality reaching up to 50% at two years in some cohorts [3], a higher rate of re-hospitalization at 30 days [4], and an increase in management costs [5].

However, the definition of this entity remains controversial. Depending on the study, the biological markers used and the populations, the prevalence of type 2 infarction varies from 2% to 58% [6]. Despite multiple attempts based on different troponin levels [7], combination of multiple biological markers [8] or clinical-biological scores [9], there is currently no standardization of the diagnosis or reliable diagnostic test for type 2 infarction. This difficulty lies with the definition of the imbalance in the oxygen supply/demand. Two main diagnostic approaches have emerged in the literature: one relies on subjective adjudication by expert review of medical records [10-12], while the other is based on the application of objective criteria derived from predefined clinical and biological parameters [13,14]. The most detailed objective criteria were described by Saaby et al. [15], notable for including the highest number of clearly defined clinical thresholds and quantified parameters to characterize oxygen supply-demand imbalance. Although their reproducibility has not been investigated. Finally, even if there is a proven imbalance of O2 in the myocardium, the stratification of the actual ischemic risk of these patients is complex. Indeed, coronary angiography is rarely performed due to comorbidities or the risk of bleeding [16]. Assuming that patients with heart disease or coronary lesions are more likely to have type 2 ACS in circumstances of myocardial stress, it would make sense that patients with no cardiological history with type 2 infarction would be more likely to have underlying heart disease or hitherto unknown coronary lesions. However, the occurrence of a type 2 infarction in this population has never been specifically studied.

Our study aimed to document the characteristics and management of patients with no history of heart disease presenting with type 2 myocardial infarction in the emergency department, and to investigate the impact of on-site coronary angiography availability on diagnosis and management using objective criteria selected for their potential reproducibility in clinical practice.

## Materials and methods

Population

**Figure 1:**
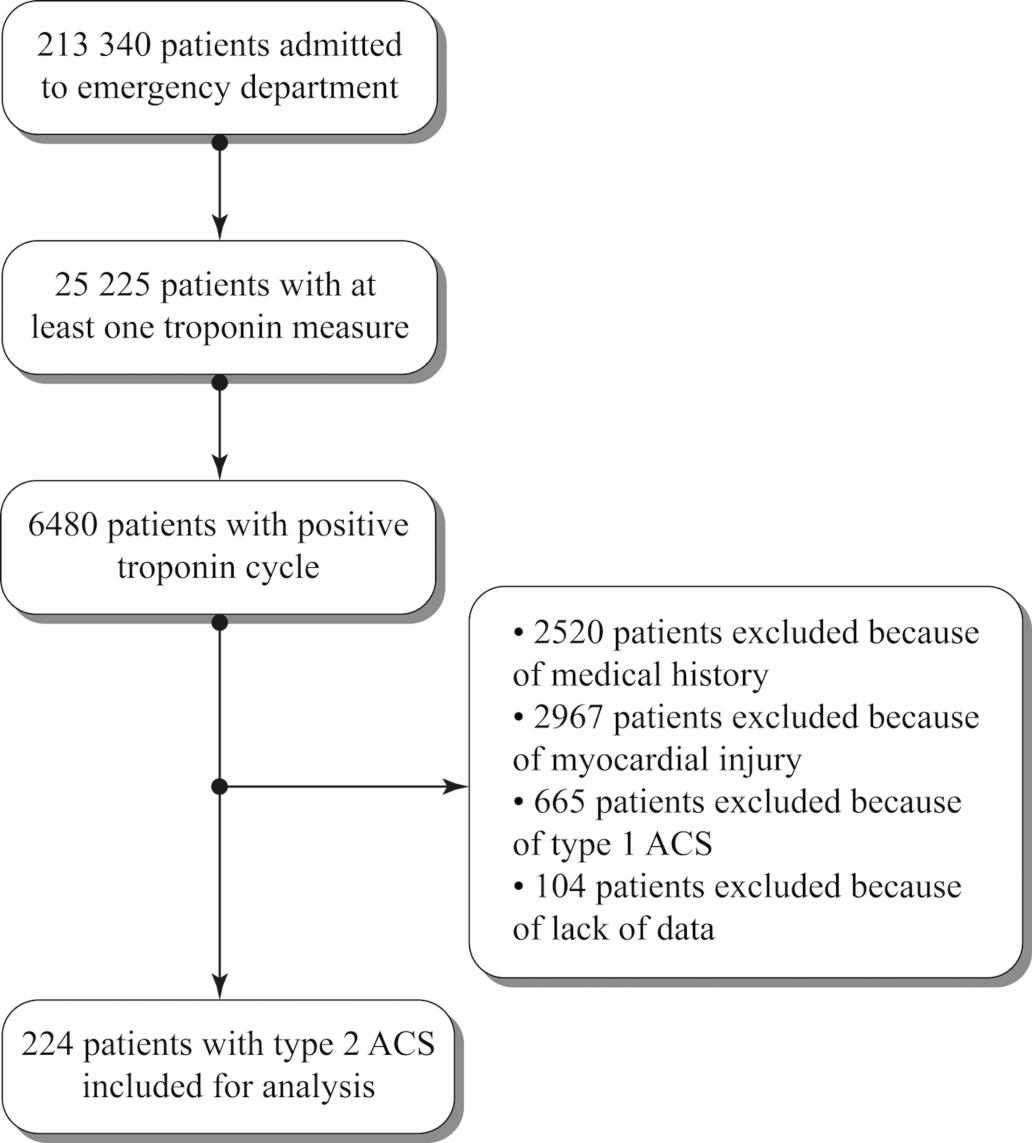
Flowchart ACS: acute coronary syndrome

The study population was obtained by extraction of 38,760 assays of high-sensitivity troponins (hs) performed on 25,225 patients out of 213,340 patients admitted to the emergency departments of the University Hospital Center of Reims, France, with a coronary angiography facility (Cath Lab center) and the Hospital Center of the city of Charleville-Mézières, France, without a coronary angiography facility (No Cath Lab center), between January 1, 2018 and December 31, 2019. A total of 6,480 patients with a positive set of troponin measurements were selected. The review of the medical records was then carried out in chronological order of arrival at the emergency room. In the case of multiple admissions for the same patient, the selected admission was the first one that met the inclusion criterion (admitted to the emergency department and a positive set of troponin measurements) without exclusion criteria. The exclusion criteria were any history of heart disease (ischemic or not), heart failure or atrial fibrillation (AF), and situations that did not meet the diagnostic criteria for type 2 ACS. Thus, 2,520 patients with a history of heart disease, 2,967 patients with a diagnosis of myocardial injury, and 665 with a diagnosis of type 1 ACS were excluded. A further 104 patients were excluded due to missing data, allowing us to obtain our population of 224 patients. This approach is outlined in the flowchart (Figure 1).

Biological testing

The troponin test kit used in the center with a Cath Lab was the "Elecsys® troponin T hs" test from ROCHE Diagnostics (Basel, Switzerland), which allowed determination of troponin T hs by an electrochemiluminescence method on a "Cobas e602" immunoassay system. The limit of detection for this test was 5 ng/L. The limit for the 99th percentile of a normal population was 14 ng/L. The coefficient of variation was 10%. The test was considered positive at a concentration >14 ng/L [17]. The troponin test kit used in the center without a Cath Lab was the "ARCHITECT STAT High-Sensitivity Troponin-I" from ABBOTT Laboratories (Chicago, IL, USA), which allowed determination of troponin I hs by an immunological method on an "ARCHITECT iSystem" immunoassay system. The limit of detection for this test was between 1.2 and 1.9 ng/L. The limit for the 99th percentile of a normal population was 16 ng/L in women and 34 ng/L in men. The coefficient of variation was 10%. The test was considered positive with a concentration >34 ng/L [18].

Since each center used a different assay, thresholds specific to each test were applied according to the ESC article on the proper use of troponin [19]. A set of troponin measurements was considered positive when the troponin level after 3 hours (H3 troponin) exhibited an increase of more than 50% if the initial troponin (H0) was negative, or if H3 troponin exhibited a variation of more than 20% when H0 troponin was positive. No harmonization of absolute values was performed, as clinical decisions and inclusion criteria were based on dynamic changes and threshold-based interpretation in routine practice.

In the case of a single measurement in the emergency department, any positive value led to an examination of the medical record. A second value was then sought in the care suites. If none was found, the patient was excluded.

Diagnostic criteria for type 2 ACS

The diagnosis of type 2 ACS was a posteriori adjudicated by a single reviewer through medical record review based on the definition of the ESC. It’s namely the combination of troponin kinetics, ischemic sign (clinical symptoms of ischemia, electrocardiographic changes indicating new ischemia such as changes in the ST segment or T wave, the appearance of a left branch block, the development of a Q wave at the ECG, an image suggestive of a new loss of myocardial viability or a new abnormality of segmental kinetics of the myocardium) and a context of mismatch between the oxygen supply and demand in the myocardium.

To define the mismatch, we used the formal criteria described by Saaby [15]. Anemia was defined as a concentration of less than 5.5 mmol/L or 89 g/L for a man, and less than 5.0 mmol/L or 80 g/L for a woman. Shock was defined as a systolic blood pressure below 90 mmHg associated with signs of organ dysfunction, such as metabolic acidosis, hypoxia (partial oxygen pressure below 60 mmHg), oliguria (urine output less than 30 mL/h for at least 3 hours), or encephalopathy. Bradycardia requiring medical treatment or electro-training was defined as a cause of mismatch. Coronary embolism was identified in patients with embolic risk factors, such as left heart endocarditis, intra-cardiac thrombus, documented deep vein thrombosis, patent foramen ovale, or interatrial communication. Respiratory failure was defined as an arterial oxygen tension below 60 mmHg associated with clinical signs of acute respiratory distress lasting more than 20 minutes. Ventricular tachycardia (VT) lasting more than 20 minutes and supraventricular tachycardia (SVT) lasting more than 20 minutes with a heart rate greater than 150 beats per minute were also defined as a cause of mismatch. Acute hypertensive pulmonary edema (AHPE) was defined as arterial hypertension greater than 160 mmHg associated with signs of AHPE and requiring diuretics or nitrate therapy. Finally, arterial hypertension (AHT) greater than 160 mmHg associated with left ventricular hypertrophy on ECG or echocardiography was included.

Data collected

All data were extracted from electronic medical records, which included all local clinical information and scanned external reports. We collected baseline characteristics (age, sex, BMI calculated from height and weight) and medical history (hypertension, diabetes, hyperlipidemia, current or past smoking, family history of cardiovascular disease) for each patient based on what was reported in the medical record. The electric presentation at the electrocardiogram (ECG) was based on the clinician’s interpretation reported in medical records. Glomerular filtration rate (GFR) at admission was calculated using the Cockcroft formula and creatinine collected at the same time as the first troponin.

We reviewed all available information in the medical record from the time of admission through the rest of the hospitalization, including any documentation added at a later stage. We noticed the mention of a request for cardiological consultation due to the positive set of troponin measurement. We found if an ischemic test was recommended during the initial hospitalization, if it was realized or not, and for which reason. If an ischemic test was realized, we consider it as invasive (coronarography) or non-invasive (stress ECG, stress echocardiography, positron emission tomography (PET) perfusion imaging). For the No Cath Lab center, an invasive test was possible with a transfer to the Cath Lab center or a private hospital in the region. We use the coronarography report to identify the presence of a culprit lesion (defined as ≥70% stenosis), the number of diseased vessels - classified as mono-, bi-, or tri-troncular depending on the number of major coronary vessel involved (LAD, LCx, or RCA) - any angioplasty performed with the corresponding cumulative stent length, and any indication for coronary artery bypass grafting.

Finally, we searched all the records for a diagnosis or an explanation retained by the clinicians to describe the association or troponin kinetics, ischemic signs and circumstances of O2 imbalance that led us to adjudicate the diagnosis of type 2 ACS.

Concerning missing data, any clinical history not mentioned in the medical record was considered absent. The absence of mention of a test or consultation was interpreted as not having been performed. Similarly, if no explanation was documented for a diagnostic or therapeutic decision (e.g., lack of ischemic investigation), it was recorded as “no justification provided”.

Ethical considerations

This work was a retrospective register based on medical records. In accordance with French law, it depends on the reference methodology “MR-004”. It requires the declaration of the use of medical data to the institution in possession of the data of the patients concerned. The declaration had to include a summary of the study and the means used to ensure data protection. This declaration was submitted to the Robert Debre University Hospital Center in Reims before the use of the database began. For the data of the second center, an agreement authorizing the use of these was signed between the University Hospital of Reims and the Hospital of Charleville-Mézières. Only the principal investigator had access to the data, which were fully pseudonymized prior to analysis.

Statistics

The study was designed as a retrospective, descriptive, and comparative analysis. Continuous variables were presented as mean ± standard deviation. Comparison performed by t-test for normally distributed variables and compared by Wilcoxon rank-sum tests for non-normally distributed variables. The distribution was assessed using histogram distribution, density curves, and QQ-plots. Categorical variables were presented as percentage and frequency compared by chi-squared test or Fisher's exact test as appropriate.

To explore the elements that influence the realization of coronarography, we conducted a multivariate analysis using a logistic regression that included all variables with a p-value < 0.20 for univariate analysis. We chose to binarize causes into “cardiac” and “non-cardiac” categories assuming that cardiac etiologies are more likely to prompt ischemic investigation, whereas non-cardiac causes often lead to conservative management. Moreover, we expected a small number of coronary angiographies, and multiplying variables would have made the model unstable.

For the analyses, a two-sided P-value of 0.05 or below was considered statistically significant. The statistical tests were performed with "SPSS Statistics" version 25 software (IBM Corp., Armonk, NY, USA).

## Results

Characteristics of the population

The main basic characteristics of our cohort are summarized in Table 1. The mean age is 76 years (+/- 14). We find that most of the patients are men (121, 54.0%) with hypertension (139, 62.1%), overweight (body mass index, BMI = 26.9 +/- 6.7) and have an average moderately impaired left ventricular ejection fraction (LVEF) at 45% (+/- 16). Few patients (31, 13.8%) were known to have a history of renal failure, whereas there was a moderately altered glomerular filtration rate (GFR) at the time of troponin assay (GFR = 58 mL/min). The ECG presentation was in the form of a non-ST Elevation Myocardial Infarction (NSTEMI) for 216 (96.4%) patients. The comparison between the two centers showed two very similar populations that differed only in the presence of a history of renal failure (p = 0.027), troponin peak (p < 0.001), and in the distribution of LVEF (p = 0.018).

**Table 1:** Baseline characteristics *p < 0.05; Cath Lab: catheterization laboratory, SD: standard deviation, BMI: body mass index, GFR: glomerular filtration rate, NSTEMI: non-ST-segment elevation myocardial infarction, LVEF: left ventricular ejection function

Variable	All (n=224)	No Cath Lab (n=87)	Cath Lab (n=137)	p-value
Age, years (± SD)	76 (14)	75 (14)	76 (15)	0.477
Male sex, no. (%)	121 (54.0)	52 (59.8)	69 (50.4)	0.173
Risk factors				
Hypertension, no. (%)	139 (62.1)	50 (58.8)	89 (65.0)	0.393
Diabetes, no. (%)	60 (26.8)	18 (21.2)	42 (30.7)	0.161
Hyperlipidemia, no. (%)	50 (22.3)	19 (22.4)	31 (22.6)	1.000
Smoking, no. (%)	66 (29.5)	28 (32.9)	38 (27.7)	0.451
Family history, no. (%)	18 (8.0)	8 (9.4)	10 (7.3)	0.618
BMI, (± SD)	26.9 (6.7)	26.1 (7.1)	27.5 (6.3)	0.168
Known chronic kidney injury, no. (%)	31 (13.8)	6 (7.1)	25 (18.2)	0.027^*^
GFR at admission, mL/min (± SD)	58 (29)	58 (29)	58 (29)	0.984
NSTEMI, no. (%)	216 (96.4)	82 (94.3)	133 (97.1)	0.361
Troponin peak, ng/L (± SD)	102 (49-416)	305 (87-1397)	77 (39-157)	< 0.001^*^
LVEF, % (± SD)	45 (16)	44 (16)	46 (15)	0.436
LVEF Class				0.018^*^
Unknown	50 (22.3)	10 (11.5)	40 (29.2)	
Preserved (> 50%)	93 (41.5)	41 (47.1)	52 (38.0)	
Mild (40-50%)	20 (8.9)	10 (11.5)	10 (7.3)	
Reduced (< 40%)	61 (27.2)	28 (29.9)	35 (25.5)	

Etiologies of the O2 imbalance

**Figure 2:**
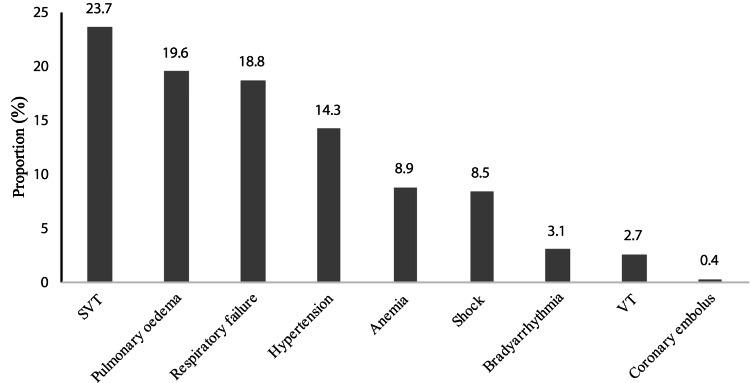
O2 mismatch etiology rate SVT: supraventricular tachycardia, VT: ventricular tachycardia

The causes of the mismatch between myocardial oxygen supply and demand in our cohort are presented in Figure 2. The most common cause was supraventricular tachycardia, which was responsible in 52 (23.7%) cases. This was followed by AHPE and respiratory failure at roughly equal frequencies for 44 (19.6%) and 42 (18.8%) patients, respectively. Hypertensive flare-up accounted for 32 (14.3%) cases. Anemia and shock were implicated in 20 (8.9%) and 19 (8.5%) patients, respectively. Bradycardia for seven (3.1%) cases, ventricular tachycardia for six (2.7%) cases, and coronary embolism for one (0.4%) case were the least common causes. For all these causes of O2 imbalance, there was no significant difference between the two populations (p > 0.05).

Clinician’s diagnosis

**Figure 3:**
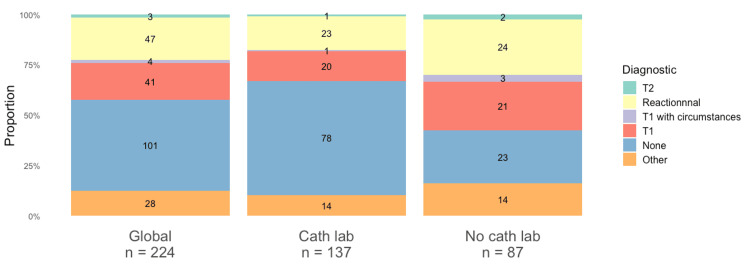
Physician's diagnosis T1: type 1 acute coronary syndrome, T2: type 2 acute coronary syndrome

The diagnoses made by the clinicians are presented in Figure 3. The diagnosis of type 2 ACS was retained by the clinicians in only three (1.3%) patients. There were four (1.8%) patients where the diagnosis of type 2 was not formally retained, but the clinicians concluded to an ACS secondary to an imbalance in the O2 intake/consumption by the myocardium. In most cases, 105 (46.9%) patients, no diagnosis was mentioned to explain the troponin change associated with acute myocardial suffering. In 47 patients (21.0%), clinicians described the troponin elevation as “reactional,” due to a suspected imbalance between oxygen supply and demand. However, despite the presence of ischemic signs, the diagnosis of acute coronary syndrome was not retained. Conversely, 41 patients (18.3%) were diagnosed with type 1 ACS, without consideration of the oxygen imbalance that was objectively present. Finally, all the remaining non-categorizable diagnoses evoked the notion of acute myocardial ischemia related to a certain degree to a triggering factor.

No statistical test could be carried out given the small numbers in each category. However, there was a higher proportion of patients without a diagnosis in the Cath Lab center (78, 59.9%) than No Cath Lab center (23, 26.4%). Their diagnostics were in favor of a higher rate of reactional troponin movements [24 (27.6%) versus 23 (16.8%)] and type 1 ACS [21 (24.1%) versus 20 (14.6%)] in the No Cath Lab center.

Explorations

Results of explorations are detailed in Table 2. In 180 (80.4%) cases, we found in medical records a request for cardiological consultation by the emergency physician. This consultation led to a theoretical indication of exploration for 87 (38.8%) patients, but was effective for 66 (29.5%) patients during the hospitalization. Among the patients who did not undergo an ischemic test during hospitalization, 118 (74.7%) had no clear justification documented in their medical records. When a justification was provided, it was mainly due to a high level of comorbidities in 24 (15.2%) patients. Exploration during the course of the event was infrequent for 12 (5.4%) patients, and was carried out at the physician's discretion in nine patients, corresponding to 75% of the delayed test. When the exploration was carried out, it was most often invasive with a coronarography for 67 patients corresponding to 84.8% of patients with an exploration. It then highlighted significant coronary lesions in 43 patients, corresponding to 64.2% of those who underwent this test. Of these patients with a culprit lesion, 31 (72.1%) underwent revascularization. Six patients had a coronary artery bypass grafting and 25 patients had an angioplasty with a mean cumulative stent length of 35.2 cm (+/- 18.6). Non-invasive explorations, which were less frequent (12 patients or 15.2% of patients with an exploration), were negative for 11 patients, corresponding to 91.7% of them.

**Table 2:** Details of investigations and diagnostic course *p < 0.05, Cath Lab: catheterize laboratory, ACS: acute coronary syndrome

	All (n=224)	No Cath Lab (n=87)	Cath Lab (n=137)			p-value
Request for cardiological consultation, no. (%)	180 (80.4)	73 (83.9)		107 (78.1)	0.306	
Recommended test during hospitalization, no. (%)	87 (38.8)	41 (47.1)		46 (34.1)	0.067	
Test performed during hospitalization, no. (%)	66 (29.5)	32 (36.8)		34 (24.8)	0.071	
Test delayed, no. (%)	12 (5.4)	1 (1.1)		11 (8.0)	0.107	
Invasive test, no. (%)	67 (29.9)	30 (34.5)		37 (27.0)	0.295	
No culprit lesion, no. (%)	24 (35.8)	11 (36.7)		13 (35.1)	0.498	
Monotroncular lesion, no. (%)	13 (19.4)	8 (26.7)		5 (13.5)		
Bitroncular lesion, no. (%)	18 (26.9)	7 (23.3)		11 (29.7)		
Tritroncular lesion, no. (%)	12 (17.9)	4 (13.3)		8 (21.6)		
Revascularization, no. (%)	31 (13.8)	12 (13.8)		19 (13.8)	0.987	
Stent length, cm (+/- SD)	35.2 (18.6)	35.4 (19.9)		35.1 (18.4)	0.967	
Non-invasive test, no. (%)	12 (5.4)	3 (3.4)		9 (6.6)	0.376	
Negative, no. (%)	11 (91.7)	3 (100.0)		8 (88.9)	1.000	
Positive, no. (%)	1 (8.3)	0 (0.0)		1 (11.1)		
Patients without ischemic test during hospitalization, no. (%)	158 (70.5)	55 (63.2)		103 (75.2)	0.071	
Reason for no test during hospitalization						
High comorbidity burden, no. (%)	24 (15.2)	11 (20.0)		13 (12.6)		
Patient's death, no. (%)	12 (7.6)	5 (9.1)		7 (6.8)		
Patient's refusal, no. (%)	4 (2.5)	3 (5.5)		1 (1.0)		
None, no. (%)	118 (74.7)	36 (65.5)		82 (79.6)		
Reason for delaying test						
Recurrent ACS, no. (%)	2 (16.7)	1 (100)		1 (9.1)		
Patient's choice, no. (%)	1 (8.3)	0 (0.0)		1 (9.1)		
Physician's choice, no. (%)	9 (75.0)	0 (0.0)		9 (81.8)		

The management between the Cath Lab and No Cath Lab centers seems to be overlapping. There was no significant difference regarding contacts with cardiologists [73 (78.1%) patients in Cath Lab Center versus 107 (83.9%) patients in No Cath Lab Center, P = 0.306] or patients who underwent invasive exploration [37 (27.0%) patients in Cath Lab Center versus 30 (34.5%) patients in No Cath Lab Center, P = 0.295], or patients with revascularization [12 (13.8%) patients in Cath Lab Center versus 19 (13.8%) patients in No Cath Lab Center, P = 0.987] or even patients with delaying test [11 (8.0%) patients in Cath Lab Center versus 1 (1.1%) patient in No Cath Lab Center, P = 0.107].

Stratification of ischemic risk

Lastly, we studied the difference between patients explored and patients not explored by an invasive method (Table 3). The patients who underwent a coronarography were younger [70 years old versus 78 years old, P < 0.001], more often men [47 (70.1%) versus 74 (47.1%), P = 0.002], more frequent smokers [29 (43.9%) versus 37 (23.7%), P = 0.004], and tended to have a family history of cardiovascular disease [14 (21.2%) versus 4 (2.6%), P < 0.001]. Patients with coronarography had better GFR at the time of the troponin measurement (68 mL/min versus 54 mL/min, P = 0.030). The ECG presentation was less likely to be an NSTEMI [61 (91%) versus 155 (98.7%) in patients without coronarography, P = 0.005]. The distribution of LVEF class differed (P = 0.002). Patients undergoing invasive exploration had a lower proportion of unknown LVEF [5 (7.5%) vs. 45 (28.7%)] and a higher proportion of reduced LVEF [25 (37.3%) vs. 36 (22.9%)].

**Table 3:** Invasive test characteristics *p < 0.05, Cath Lab: catheterize laboratory, SD: standard deviation, BMI: body mass index, GFR: glomerular filtration rate, NSTEMI: non-ST-segment elevation myocardial infarction, LVEF: left ventricular function, SVT: supraventricular tachycardia, VT: ventricular tachycardia

Characteristics	Invasive test (n=67)	Without invasive test (n=157)	p-value
Age, years (± SD)	70 (13)	78 (14)	< 0.001^*^
Male sex, no. (%)	47 (70.1)	74 (47.1)	0.002^*^
Risk factors			
Hypertension, no. (%)	38 (57.6)	101 (64.7)	0.363
Diabetes, no. (%)	21 (31.8)	39 (25.0)	0.323
Hyperlipidemia, no. (%)	16 (24.2)	34 (21.8)	0.726
Smoking, no. (%)	29 (43.9)	37 (23.7)	0.004^*^
Family history, no. (%)	14 (21.2)	4 (2.6)	< 0.001^*^
BMI, (± SD)	26.9 (4.8)	26.9 (7.5)	0.948
Known chronic kidney injury, no. (%)	8 (12.1)	23 (14.8)	0.676
GFR at admission, mL/min (± SD)	68 (26)	54 (29)	0.002^*^
NSTEMI, no. (%)	61 (91)	155 (98.7)	0.005^*^
Troponin peak, ng/L (± SD)	1016 (2602)	735 (2529)	0.452
LVEF Class			
Unknown	5 (7.5)	45 (28.7)	0.002^*^
Preserved (> 50%)	28 (41.8)	65 (41.5)	
Mild (40-50%)	9 (13.4)	11 (7.0)	
Reduced (< 40%)	25 (37.3)	36 (22.9)	
O2 imbalance etiology			
Anemia, no. (%)	5 (7.5)	15 (9.6)	0.799
Respiratory failure, no. (%)	6 (9.0)	36 (22.9)	0.015^*^
SVT, no. (%)	11 (16.4)	42 (26.8)	0.122
VT, no. (%)	6 (9.0)	0 (0.0)	0.001^*^
Pulmonary edema, no. (%)	20 (29.9)	26 (16.6)	0.030^*^
Shock, no. (%)	0 (0.0)	19 (12.1)	0.003^*^
Bradyarrhythmia, no. (%)	1 (1.5)	6 (3.8)	0.449
Hypertension, no. (%)	18 (26.9)	15 (9.6)	0.001^*^
Coronary embolus, no. (%)	1 (1.5)	0 (0.0)	0.299
Request for cardiological consultation, no. (%)	65 (97.0)	115 (73.2)	< 0.001^*^

The etiology of the mismatch between the myocardial oxygen supply and demand played a role in the exploration indication. Indeed, the patients with invasive exploration were more likely to present with ventricular tachycardia [6 (9.0%) versus 0 (0.0%) P = 0.001] or AHPE [20 (29.9%) patients versus 26 (16.6%) patients, P = 0.030] or a hypertensive flare-up [18 (26.9%) versus 15 (9.6%), P = 0.001]. In contrast, they were less likely to have experienced respiratory failure [6 (9.0%) versus 36 (22.9%), P = 0.015] or shock [0 (0.0%) versus 19 (12.1%), P = 0.003]. The other etiologies did not show a significant difference. Finally, the patients who had undergone coronarography significantly received more consultation with a cardiologist [65 (97.0%) versus 115 (73.2%), P < 0.001].

**Figure 4:**
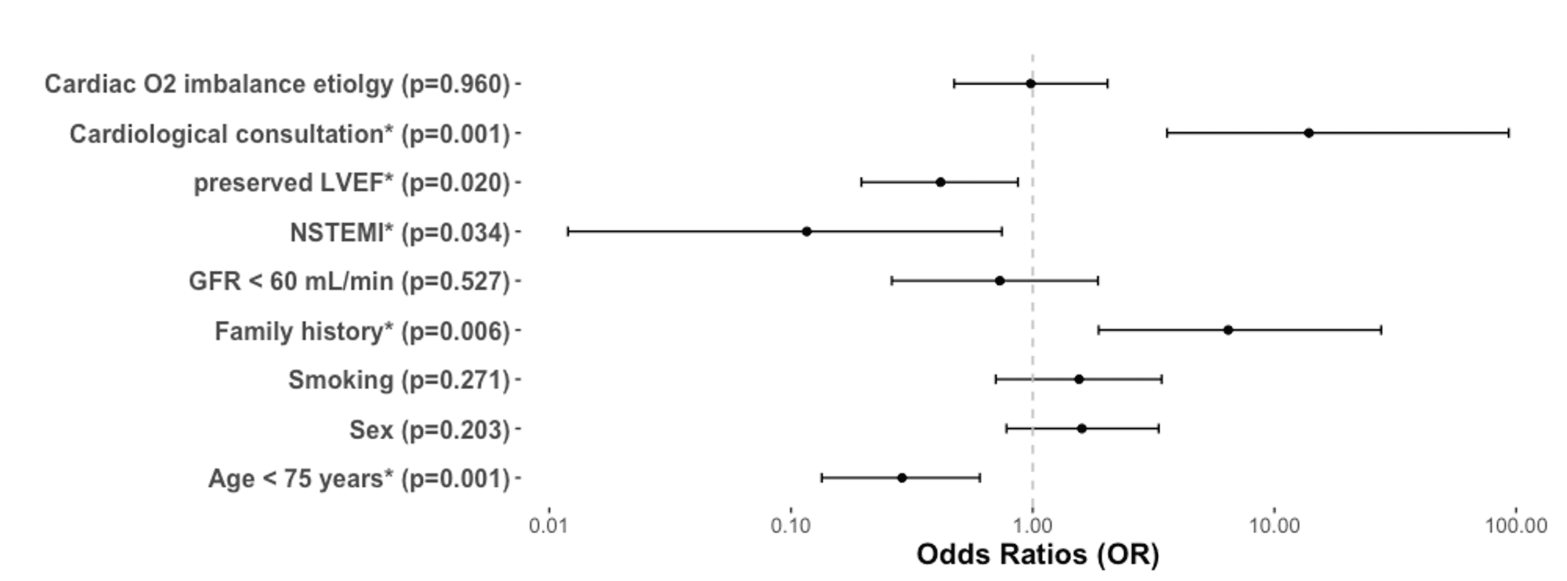
Multivariate analysis *p < 0.05; GFR: glomerular filtration rate, NSTEMI: non-ST-segment elevation myocardial infarction, LVEF: left ventricular ejection function

The multivariate analysis is detailed in Figure 4. A family history of vascular disease (P = 0.006) and a request for a cardiological consultation (P = 0.001) remained significantly associated with a coronarography’s realization, while an age below 75 years (P = 0.001), a preserved LVEF (P = 0.020) and an ECG presentation as NSTEMI (P = 0.034) were still significantly associated with the absence of invasive exploration.

## Discussion

Our results showed that the use of objective diagnostic criteria allowed the selection of a homogeneous population in two distinct centers. Nevertheless, the diagnosis of type 2 ACS was rarely found in the patient records. Patients without a history of heart disease were often explored, and when this was done, the exploration was frequently positive and led to revascularization. They appeared to be an interesting population to target for ischemic investigations. Surprisingly, the presence or absence of a coronary angiography facility in the center didn’t seem to significantly alter the patient's management.

Population characteristics

Our cohort was derived from an unselected population of patients consulting at the emergency room (ER) but strongly selected for the absence of a history of heart disease. The consequence is a decrease in the prevalence of type 2 ACS to 0.9%, while it oscillates between 2.4% and 8.5% in the other studies carried out on an unselected population of patients consulting at the ER [20-22]. Despite this selection, our patients were similar to those in the original Saaby cohort [15] in terms of age, sex ratio, cardiovascular risk factor profiles, and LVEF distribution. We also found substantially the same distribution of causes, with a preponderance of supraventricular tachycardia and respiratory failure. This homogeneity between our centers and the cohorts in the literature shows that the use of formal criteria to qualify the mismatch between the oxygen supply and demand allows for good reproducibility. Nevertheless, these criteria could be further improved. For instance, using age-adjusted thresholds, such as theoretical maximum heart rate, to define tachycardia would make the mismatch definition more physiologically accurate, avoiding misclassification of very young or elderly patients.

Type 2 ACS diagnosis

Our study shows a flagrant underdiagnosis of type 2 ACS, formally identified by clinicians in only 1.3% of cases. For this pathology, the concordance between the clinician's diagnosis and the subsequent diagnosis is known to be low [23]. In the SWEDEHEART registry, the prevalence of type 2 infarction selected by the clinician was 6.2% of infarctions, while it was 12.0% retrospectively identified [24]. In our cohort, this diagnostic gap was even more pronounced. In 21% of cases, clinicians documented a condition of myocardial stress, such as tachyarrhythmia or respiratory failure, as the likely cause of troponin elevation, yet did not retain a formal diagnosis of type 2 ACS. These situations were often described using vague or non-specific terms like “reactive troponin” without fulfilling the criteria for myocardial infarction in documentation. This practice may reflect diagnostic inertia, limited familiarity with type 2 ACS, or reluctance to label an ACS without ST-segment elevation or a typical presentation. Notably, diagnostic uncertainty persisted despite a high rate of cardiology consultation (80%), and nearly half of the patients (46.9%) had no explanation documented for the observed troponin rise.

The clinical consequences of such under-recognition can vary. Misclassifying type 2 ACS as type 1 may lead to overtreatment, including unnecessary dual antiplatelet therapy or early invasive strategies, while failing to diagnose type 2 at all may result in under-treatment or missed opportunities for secondary prevention.

These findings underscore the need to improve awareness among emergency physicians and cardiologists regarding troponin interpretation and the appropriate application of myocardial infarction definitions across all types. This effort may be supported by the implementation of ICD-10 codification (I21.A1, since 2017), which has led to increased recognition of type 2 ACS in United States administrative data [25].

Type 2 ACS management

Our working hypothesis was that patients with no history of heart disease would be explored more after a type 2 ACS and would have more coronary lesions. This hypothesis was confirmed. Even though the proportion of patients explored (29.9%) corresponds to the average rate found by Sandoval's meta-analysis in 2017 [26]. When compared to all the studies reported by the summary article of the same author in 2019 [27], our coronary lesion rate is among the highest, with significant lesions in 64.2% of the patients. Moreover, this exploration had practical consequences. About 72.1% of those with lesions underwent revascularization, either percutaneous (stenting) or surgical (bypass), highlighting the clinical relevance of coronarography in this population.

However, a first stratification of the ischemic risk was already carried out by the clinician. Invasive testing was not proposed for all and tended to be guided by clear clinical factors such as age, LVEF, ECG presentation and a request for cardiological consultation. All of which were significantly associated with exploration in multivariate analysis. Interestingly, other expected risk markers such as male sex, smoking, and renal function were not retained as independent predictors. In the case of renal impairment, this may reflect real-life decision-making, where clinicians adjust contrast use to reduce nephrotoxicity when coronary disease is strongly suspected, thus neutralizing its role as a limiting factor.

Regarding the cause of oxygen imbalance, univariate analysis suggested that etiologies more likely related to underlying heart disease (e.g., hypertensive crisis, arrhythmias) were associated with a higher rate of exploration, while extracardiac stressors (e.g., respiratory failure, shock) were explored less. However, these associations did not persist in multivariate analysis. This may indicate that the causal category has less weight than expected. Additionally, our binary grouping of etiologies (cardiac vs. non-cardiac) may have diluted the effect of more specific causes that could emerge with a larger sample size.

Strengths and limitations

Our interpretations are mainly limited by the retrospective nature of the study. The absence of key initial data, such as ischemic signs or sufficient information to define oxygen supply-demand imbalance, led to the exclusion of 104 patients who could not be reliably classified as type 1 ACS, type 2 ACS, or myocardial injury. The retrospective design also may have introduced imprecision that affected the consistency of diagnostic classification. In addition, differential selection bias between centers may have occurred due to differences in the structure of medical record systems. It is possible that some ischemia searches may have been carried out in other centers and do not appear in the patient files. Furthermore, although the inclusion of only patients without known heart disease allowed for a highly specific population, it also limits the generalizability of our findings to broader ACS type 2 populations, including elderly or high-risk patients. The absence of long-term follow-up data, such as mortality or readmission, precludes any conclusions regarding the prognostic impact of diagnostic or therapeutic strategies.

Despite these imperfections, the strength of our study lies in the innovative nature of the population studied. Indeed, to our knowledge, no studies have involved patients with no history of heart disease. Just one study involved only young patients with no history of coronary artery disease [11]. Even with this inclusion restriction, the number of subjects studied is at the level of previously published cohorts. It is also the only study to date to compare patient management based on the presence of a coronary angiography facility. Finally, it is one of the few studies to examine the discrepancy between the diagnosis made during care and that retained a posteriori.

A randomized controlled trial (ACT-2) is currently underway to compare coronary angiography versus medical management in patients with type 2 ACS in order to evaluate the impact of an invasive strategy in this context [28].

## Conclusions

Our study demonstrated that the use of objective criteria provides a clear, reproducible, and clinically coherent method for identifying patients with type 2 ACS, consistent with current literature. The diagnosis is rarely made by the clinician, but with frequent recourse to invasive and non-invasive coronary explorations. These results should encourage targeted awareness campaigns among emergency physicians and cardiologists in order to improve recognition of type 2 ACS. They also support the integration of objective diagnostic criteria into future clinical guidelines to promote more consistent identification and management of this underdiagnosed condition.
